# Enhancing reactive strength index through braking-phase focused verbal instruction

**DOI:** 10.3389/fspor.2026.1698649

**Published:** 2026-03-27

**Authors:** Kuniaki Hirayama, Shingo Nakai, Takafumi Kubo, Seiichiro Takei

**Affiliations:** 1 Faculty of Sport Sciences, Waseda University, Tokorozawa, Japan; 2 Faculty of Sport Science, Shizuoka Sangyo University, Iwata, Japan; 3 Graduate School of Sport Sciences, Waseda University, Tokorozawa, Japan; 4 Sendai 89ers, Sendai, Japan; 5 Teikyo University Institute of Sports Science and Medicine, Hachioji, Japan

**Keywords:** ankle hop, cueing, electromyography, plyometrics, stretch–shortening cycle

## Abstract

This study examined whether verbal instruction emphasizing force exertion during the braking phase of rebound jumps can acutely enhance the reactive strength index (RSI), a key indicator of stretch–shortening cycle performance linked to sprinting and change-of-direction ability. Seventeen physically active men without plyometric training experience were randomly assigned to an intervention (*n* = 8) or control group (*n* = 9). Both groups performed repeated rebound jumps under standard instruction. The intervention group additionally received braking-phase–focused instruction. RSI significantly increased only in the intervention group (*p* < 0.05), accompanied by greater soleus muscle preactivation before ground contact. These findings suggest that simple, targeted verbal instruction can acutely optimize neuromuscular strategies during plyometric tasks, offering coaches a practical tool for enhancing explosive performance without long-term training.

## 1 Introduction

Verbal instruction, or cueing, is a common coaching tool used to optimize athletes’ movement execution. Because human movement is generated by muscle contractions associated with muscle activity, coaches can be said to intervene in athletes’ muscle activity through verbal instruction in order to enhance motor performance. In addition to instruction, other strategies to acutely improve muscle activity and motor performance include changing grips (1, 2), limb or body position (2–4), range of motion (5), and equipment type (6), as well as providing biofeedback (7). Of these methods, verbal instruction is relatively simple. Therefore, coaches are consistently seeking better instruction. Recent studies have demonstrated that verbal instruction is not merely a general motivational tool, but that the specific content and attentional focus of instructions can substantially influence how movements are performed and affect performance outcomes. For example, variations in verbal cues have been shown to affect performance metrics and force-time characteristics in jumping and landing tasks (8, 9).

However, there is relatively little research on the changes in muscle activity and/or motor performance attributable to verbal instruction. In one study, Snyder & Leech (10) successfully increased muscle activity in the latissimus dorsi during a lat pull-down via verbal technical instruction. Although recent research has expanded the scope of verbal instruction by examining diverse types of cues and attentional strategies, to the best of our knowledge, no studies have examined the effects of verbal instruction on the stretch–shortening cycle (SSC) of the muscle–tendon unit that accompanies most human movements in sport. In particular, the acute effects of phase-specific verbal instruction targeting distinct phases of SSC actions, such as the braking phase, remain unclear.

The reactive strength index (RSI), a performance measure of SSC, is calculated as the jump height divided by the ground contact time, and it has been reported to be associated with various athletic performances such as sprinting and change of direction (11). In addition, coaches have developed acute (practice and instructions) and chronic (plyometric training) interventions to improve athletic performance. Prior works have shown that plyometric practice (12) and training (13) can shift agonist muscle activity from the propulsion to the braking phase, enhancing tendon recoil and jump performance. Yet such adaptations typically require repeated practice or long-term training.

We hypothesized that emphasizing braking-phase force through verbal instruction could elicit earlier changes in muscle activity and improve SSC-type jump performance without prolonged training. This study therefore investigated the acute effects of braking-phase focused instruction on rebound jump performance and agonist muscle activation.

## 2 Material and methods

### 2.1 Subjects

The subjects included 17 men with exercise habits but no prior plyometric training experience. Subjects were randomly assigned to the intervention group [*n* = 9; age, 21 ± 2 years (range, 19–26); height, 170.1 ± 7.1 cm; weight, 64.6 ± 8.9 kg] or control group [*n* = 8; age, 21 ± 3 years (range, 18–27); height, 173.1 ± 5.5 cm; weight, 66.1 ± 10.6 kg]. Age, height, and weight did not significantly differ between the two groups. Subjects were informed of the nature of the study and its risks and benefits, and written consent was obtained for all subjects using an institution-approved informed consent form. However, the purpose and hypotheses of the study were not disclosed until the end of the experiment because of the possibility of bias and other factors that could affect the results of the experiment. The study was approved by the Research Ethics Committee for Human Subjects at Waseda University (Approval Number: 2018-013).

### 2.2 Procedures

All subjects warmed up by cycling for 5 min on a bicycle ergometer (20 W, 60 rpm), followed by a standardized dynamic warm-up of the lower extremities. Then, the subjects performed 10 sub-maximal repeated rebound jumps (RJs) with the instruction “keep the jump height low and the ground contact time as short as possible” for both warm-up and practice of the experimental trials. After resting for 1 min, the subjects performed five repetitions of repeated RJs at maximal effort with the same instruction. During the jumps, the subjects kept their hands on their pelvis to eliminate the influence of arm swing.

Three minutes after the warm-up, the subjects were asked to stand on a force plate (strain plate, DKH, Tokyo, Japan, 600 mm × 600 mm × 65 mm), surrounded by wooden blocks (200 mm wide by 65 mm high) to prevent accidents caused by stepping off the plate, and subjects were instructed to “jump as high as possible with the shortest ground contact time.” The subjects performed five repeated RJs with maximal effort as the baseline measurement (pre). During a 3 min rest period after the baseline measurement, the two phases of a single RJ (braking and propulsion phases) were explained to the subjects in the intervention group using simple hand and body gestures to illustrate the movement direction. After confirming that the participants understood these phases, the instruction “push the ground as hard as possible during the braking phase” was given. Meanwhile, the control group was again instructed to “jump as high as possible with the shortest ground contact time.” Care was taken not to convey the purpose or hypothesis to the subjects by tone of voice or other means. After the rest period, the instructions for each group were reiterated immediately before the post-test, and the subjects then performed five repeated RJs at maximal effort.

### 2.3 Measurement

**Figure 1:**
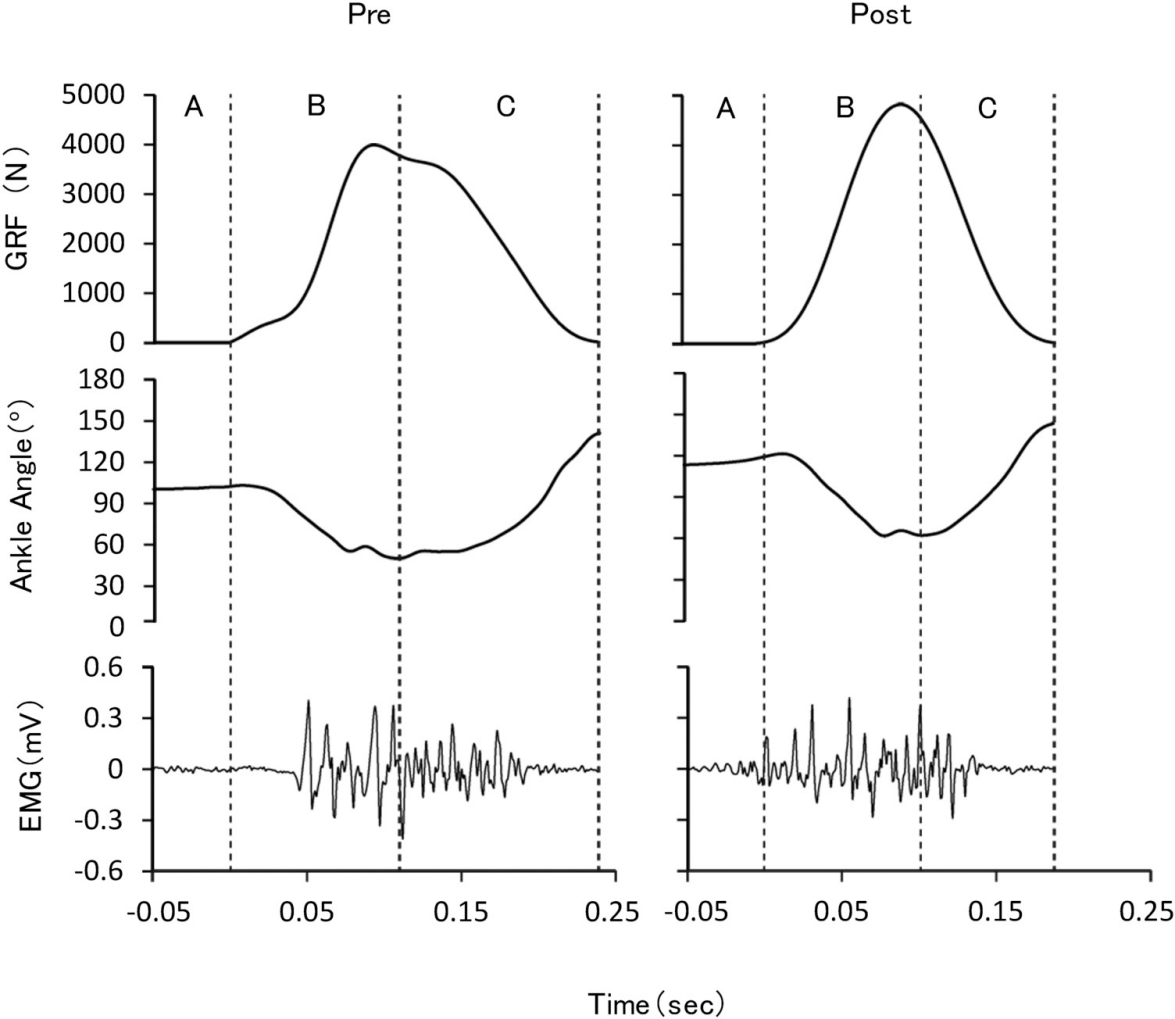
Typical example of the time course of the measured variables during rebound jumps in the first and second sets for the intervention group. GRF, ground reaction force; EMG, electromyographic activity of the soleus muscle; **(A)** preactivation phase; **(B)** braking phase; **(C)** propulsion phase.

The ground contact time and aerial time during RJs were measured using the force plate. The ankle joint angle (dorsiflexion and plantar flexion) was measured using a uniaxial angle sensor (DL-262, S&ME, Tokyo, Japan) fixed on the skin of the fibula and on the outside of the shoe. The ankle joint angle was defined as the angle between the fibula and the plantar surface, and the anatomical normal position was 90°. The angle sensor was applied prior to warm-up and calibrated using a goniometer. The muscle activity in the soleus muscle of the right foot was measured using an electromyographic (EMG) sensor (FAD-SEMG1: 4-Assist, Tokyo, Japan; input impedance, 10 GΩ; frequency response, 10–1,000 Hz). Active electrodes (20 mm apart) were attached to the lateral side of the soleus muscle belly, aligned parallel to the longitudinal axis of the muscle fibers, with the recording site located approximately between the medial femoral condyle and the medial malleolus (12, 13), in accordance with commonly used anatomical guidelines for surface EMG recordings of the lower leg muscles (14). The skin of the site was cleaned prior to electrode application. All signals were loaded at 1,000 Hz via an A/D converter (PowerLab/16sp, ADInstruments, Sydney, Australia) and synchronized using data acquisition and analysis software (LabChart8, ADInstruments, Sydney, Australia) on a personal computer (Figure 1).

### 2.4 Data analysis

LabChart8 was used for data analysis. We defined the ground contact period as the interval in which the ground reaction force was at least 10 N, and the ground contact time and aerial time were obtained. The jump height was calculated from the aerial time. In this study, the jumps with the highest RSI during five repeated jumps in both the pre and posttests were adopted in the analysis. The ankle joint angles at ground contact, maximum dorsiflexion, and takeoff were determined, and the phases before and after the time point of maximum dorsiflexion during ground contact were defined as the braking and propulsion phases, respectively. In addition to these phases, the soleus muscle EMG root mean square was obtained 50 ms before ground contact as the preactivation phase.

### 2.5 Statistical analyses

Descriptive data are presented as means and standard deviations. All data were checked for normality using the Shapiro–Wilk test. Two-way repeated-measures analysis of variance was performed to examine the change in each parameter in both groups (group × time). If the interaction was significant, a simple main effect test was performed using the Bonferroni method. Statistical significance was indicated by *p* < 0.05. Eta-squared (*η*^2^) was reported to illustrate the effect size of analysis of variance and interpreted as follows: 0.01, small; 0.06, moderate; and 0.14, large (15). For the *post hoc* test, Cohen's *d* effect sizes were calculated and interpreted as follows: 0.00–0.19, trivial; 0.20–0.59, small; 0.60–1.19, moderate; 1.20–1.99, large; 2.00–3.99, very large; and ≥4.00, nearly perfect (16).

## 3 Results

A significant interaction was found for RSI (*p* < 0.01, *η*^2^ = 0.01), with a significant increase recorded only in the intervention group (*p* < 0.001). The practical significance of the increase was small (*d* = 0.39). Although both the jump height and ground contact time, which determine RSI, were not significantly changed in the intervention group, the practical significance was higher for the ground contact time (*d* = −0.41, interpreted as small) than for the jump height (*d* = 0.19, interpreted as trivial; Table 1).

**Table 1:** Reactive strength index (RSI), jump height, and ground contact time.

Variables	Group	1st set	2nd set	Difference		
				Mean	95%CI	Cohen's d
RSI (m/s)	Intervention	1.59 ± 0.35	1.71 ± 0.36***	0.12	0.42 – 0.20	0.39
	Control	1.60 ± 0.24	1.60 ± 0.25	0.00	−0.08 – 0.08	0.01
Jump height (m)	Intervention	0.264 ± 0.037	0.270 ± 0.031	0.006	−0.019 – 0.031	0.19
	Control	0.277 ± 0.033	0.277 ± 0.026	0.000	−0.026 – 0.027	0.02
Ground contact time (s)	Intervention	0.17 ± 0.027	0.162 ± 0.022	−0.008	−0.025 – 0.008	−0.41
	Control	0.174 ± 0.016	0.175 ± 0.013	0.001	−0.017 – 0.018	0.04

*** Significant difference between the first and second sets (*p* < 0.001).

No interaction or main effect was found for the ankle joint angle at the time of ground contact, maximal dorsiflexion, or takeoff (*η*^2^ = 0.00–0.01; Table 2). Regarding the root mean square of the soleus EMG, a significant interaction was noted during the preactivation phase (*p* < 0.05, *η*^2^ = 0.03), and a significant increase was observed only in the intervention group (*p* < 0.01, *d* = 0.74 interpreted as small). Conversely, no interaction or main effect was found for the braking and propulsion phases (*η*^2^ < 0.00, Table 3).

**Table 2:** Ankle joint angles in the three phases of a rebound jump.

Variables	Group	1st set	2nd set	Difference		
				Mean	95%CI	Cohen's d
Touch down (°)	Intervention	110 ± 11	114 ± 13	3	−3 – 9	0.25
	Control	118 ± 12	117 ± 9	−2	−8 – 5	−0.13
Maximal dorsiflexion (°)	Intervention	63 ± 9	63 ± 5	0	−4 – 5	0.05
	Control	66 ± 12	64 ± 11	−2	−7 – 3	−0.23
Takeoff (°)	Intervention	135 ± 7	135 ± 8	−1	−6 –5	−0.06
	Control	140 ± 11	141 ± 12	0	−5 – 6	0.01

**Table 3:** Root mean square values of the electromyographic (EMG) activity of the soleus muscle in the three phases of a rebound jump.

Variables	Group	1st set	2nd set	Difference		
				Mean	95%CI	Cohen's d
Pre-activation phase (mV)	Intervention	0.043 ± 0.025	0.065 ± 0.038**	0.022	0.003 – 0.042	0.74
	Control	0.049 ± 0.025	0.051 ± 0.030	0.001	−0.020 – 0.022	0.05
Braking phase (mV)	Intervention	0.119 ± 0.036	0.122 ±0.033	0.003	−0.027 – 0.033	0.1
	Control	0.122 ± 0.032	0.118 ± 0.020	−0.003	−0.035 – 0.029	−0.10
Propulsive phase (mV)	Intervention	0.090 ± 0.026	0.086 ± 0.027	−0.004	−0.027 – 0.019	−0.17
	Control	0.082 ± 0.025	0.080 ± 0.014	−0.001	−0.026 – 0.023	−0.06

** Significant difference between the first and second sets (*p* < 0.01).

## 4 Discussion

The primary finding of this study was that verbal instruction emphasizing force exertion during the braking phase of rebound jumps led to an acute improvement in reactive strength index (RSI) among individuals without plyometric training experience. This improvement was accompanied by a significant increase in soleus preactivation, whereas muscle activity during the braking and propulsion phases remained unchanged. Taken together, these results suggest that a simple verbal cue can modify neuromuscular control strategies and enhance SSC-based performance, even in the absence of prior plyometric training.

Our hypothesis was partially supported. We expected that braking-phase instruction would directly increase EMG activity during the braking phase, but this was not observed. Instead, the soleus exhibited greater preactivation, beginning approximately 50 ms before ground contact. This finding aligns with previous reports that preactivation is critical for optimizing SSC performance because it allows muscle–tendon units to develop stiffness in anticipation of impact and thereby develop force more effectively during the short ground contact period (17). Increased soleus preactivation may contribute to a stiffer ankle joint at ground contact, which has been shown to be a key mechanical determinant of efficient SSC performance (18). In this context, the instruction likely prompted participants to “prepare” earlier for impact, leading to improved RSI despite no measurable change in braking-phase EMG activity.

RSI improved even though jump height and ground contact time did not individually change significantly. This discrepancy may be attributable to limited statistical power due to the relatively small sample size. Nevertheless, effect size estimates suggested that reductions in ground contact time contributed more to RSI improvement than did changes in jump height. This pattern is consistent with prior literature demonstrating that minimizing ground contact time is a key determinant of SSC efficiency and overall athletic performance (12). Future studies with larger samples are warranted to confirm these trends and to establish the robustness of the observed effects.

From a methodological and theoretical perspective, the interpretation of changes in RSI warrants careful consideration, as RSI is a composite metric influenced by multiple interrelated performance variables. Recent work by Krzyszkowski et al. (19) demonstrated that phase-specific contributors to jump performance can differentially influence global outcome measures, and that meaningful changes in RSI-related characteristics may not always be reflected by statistically significant alterations in individual variables such as jump height or ground contact time, particularly in studies with limited sample sizes. In this context, the present findings suggest that braking-phase–focused verbal instruction may have elicited subtle yet functionally relevant modifications in neuromuscular strategy that were captured by RSI but not by its individual components. This interpretation is further supported by the observed increase in soleus preactivation, which likely contributed to improved force development during the early ground contact period. Thus, even in the absence of significant changes in isolated kinematic or temporal variables, RSI appears sensitive to instruction-induced alterations in SSC function.

From a practical perspective, our findings highlight the potential of carefully worded verbal instructions in coaching. Cueing athletes to focus on braking-phase force can acutely enhance RJ performance and may also transfer to other SSC-dominant tasks such as sprinting, cutting, and depth jumping. Importantly, these results underscore the need to standardize instruction when using RJs as a diagnostic tool. Because RSI is influenced by both physical capacity and technical execution, inconsistent or vague instructions may introduce unwanted variability and reduce the validity of interindividual comparisons. Indeed, previous research has suggested that insufficient attention to the exact wording and interpretation of task instructions can substantially influence movement execution and performance outcomes, potentially contributing to variability and inconsistencies across studies (20).

The present findings also have implications for understanding the mechanisms of motor learning. Previous studies have shown that long-term plyometric training shifts agonist muscle activity toward the braking phase and away from the propulsion phase, reflecting an optimization of muscle–tendon interaction (13). In contrast, the present study demonstrates that acute modifications in muscle activation strategy can be elicited by a single verbal cue. Although the underlying mechanisms likely differ—long-term adaptations involving reduced Golgi tendon inhibition (21) and altered neural drive vs. acute cue-induced modulation—both approaches highlight the plasticity of neuromuscular control in SSC tasks. It remains to be determined whether repeated exposure to braking-phase instructions accelerates the acquisition of training-induced adaptations.

Several limitations should be acknowledged. First, EMG was recorded only from the soleus muscle. While the soleus is a major contributor to ankle stiffness and RJ performance (18, 22), changes in other muscles such as the gastrocnemius, tibialis anterior, or knee extensors could also play important roles. Previous studies have shown that tibialis anterior muscle activity does not acutely change following short-term practice (12), whereas long-term plyometric training can induce adaptations in antagonist muscle activation patterns (13), suggesting that acute instruction and chronic training may differentially affect synergistic and antagonist muscles. Second, the sample consisted exclusively of physically active men without plyometric training experience; thus, the generalizability of these findings to women, trained athletes, or clinical populations remains uncertain. Third, the relatively small sample size may have limited the detection of significant changes in jump height and ground contact time.

In conclusion, this study provides evidence that a simple verbal instruction emphasizing braking-phase force can acutely enhance RSI by promoting soleus preactivation in untrained individuals. These findings not only support the practical use of verbal cues in coaching but also emphasize the need for standardized instructions when assessing SSC performance. Future research should explore the longitudinal effects of braking-phase instruction, its applicability to trained athletes, and its potential to augment or accelerate adaptations achieved through plyometric training.

## Data Availability

The raw data supporting the conclusions of this article will be made available by the authors, without undue reservation.
